# PERCEPTIONS ON THE IMPORTANCE OF VACCINATION AND VACCINE REFUSAL IN A MEDICAL SCHOOL

**DOI:** 10.1590/1984-0462/;2019;37;1;00008

**Published:** 2018-08-09

**Authors:** Amanda Hayashida Mizuta, Guilherme de Menezes Succi, Victor Angelo Martins Montalli, Regina Célia de Menezes Succi

**Affiliations:** a Faculdade de Medicina e Odontologia São Leopoldo Mandic, Campinas, SP, Brasil.

**Keywords:** Vaccination, Vaccination refusal, Physicians, Students, medical, Ethics, Vacinação, Recusa de vacinação, Médicos, Estudantes de medicina, Ética

## Abstract

**Objective:**

To identify the perception of medical students and physicians on the importance of vaccination and the risks of vaccine refusal.

**Methods:**

Cross-sectional study with application of questionnaires about vaccines, vaccine refusal and its repercussions on public and individual health. A sample of 92 subjects was selected from a private medical school: group 1 (53 students from first to fourth grades) and group 2 (39 physicians). Data collected were tabulated in the Microsoft Excel Program and analyzed by Fisher’s exact test.

**Results:**

Both groups considered the National Immunization Program reliable and recognized the importance of vaccines, but 64.2% of students and 38.5% of physicians are unaware of the vaccine-preventable infectious diseases in the basic immunization schedule. Most of the interviewees had a personal vaccine registry, but not all had received the 2015 *influenza* vaccine. Both groups had known people who refused vaccines for themselves or for their children (respectively, 54.7 and 43.3% of students and 59.0 and 41.0% of physicians). The total of 48.7% of physicians had already assisted vaccine refusers. Appointed causes of vaccine refusal were: fear of adverse events, philosophical and religious reasons and lack of knowledge about severity and frequency of diseases. Ethical aspects of vaccine refusal and legal possibilities of vaccine requirements for children are not consensus.

**Conclusions:**

Medical students and doctors are not adequately vaccinated and have queries about the vaccination schedule, vaccine safety and vaccine refusal. Improving these professionals’ knowledge is an important strategy to maintain vaccine coverage and address vaccine refusal ethically.

## RESUMO

**Objetivo:**

Identificar a percepção da importância das vacinas e os riscos da recusa vacinal entre alunos de Medicina e médicos.

**Métodos:**

Estudo transversal realizado por meio da aplicação de questionários sobre vacinas, recusa vacinal e suas repercussões acerca da saúde pública e individual. A amostra, de 92 sujeitos, foi selecionada numa escola privada de Medicina: grupo 1 (53 estudantes do primeiro ao quarto ano) e grupo 2 (39 médicos). Os dados colhidos foram tabulados no programa Microsoft Excel e analisados estatisticamente com o teste exato de Fisher.

**Resultados:**

Os dois grupos consideram o Programa Nacional de Imunizações confiável e reconhecem a importância das vacinas, mas 64,2% dos estudantes e 38,5% dos médicos desconhecem o número de doenças infecciosas evitáveis pelas vacinas no calendário básico. A maioria dos entrevistados possuía carteira de vacinas, mas nem todos receberam vacina *influenza* 2015. Conheciam pessoas que recusavam vacinas e/ou recusavam vacinar seus filhos (respectivamente, 54,7 e 43,3% dos estudantes e 59,0 e 41,0% dos médicos). Dos médicos, 48,7% já atenderam pacientes que se recusaram a receber vacinas. Consideram causas de recusa vacinal: medo de eventos adversos, razões filosóficas, religiosas e desconhecimento sobre gravidade e frequência das doenças. Aspectos éticos da recusa vacinal e possibilidades legais de exigir vacinas para crianças não são consenso.

**Conclusões:**

Alunos de Medicina e médicos não se vacinam adequadamente, apresentam dúvidas sobre calendário vacinal, segurança das vacinas e recusa vacinal. Melhorar sua capacitação é importante estratégia para manter as coberturas vacinais e abordar a recusa vacinal de forma ética.

## INTRODUCTION

Despite being a cost-effective health investment with huge impact on health, avoiding millions of deaths per year and increasing life expectancy,^1^ ^,^ ^2^ vaccines are not universally accepted. As the number of vaccines made available increased, as well as their use in public health programs, so did the number of people and groups reporting concerns regarding safety and the actual need to take them. Parents, caregivers, patients and health professionals themselves are part of these groups.^3^ ^,^ ^4^ ^,^ ^5^ ^,^ ^6^ ^,^ ^7^

In Brazil, the National Immunization Program (NIP) relies on the credibility and respectability by the population and the scientific community, and vaccine coverage has been higher than 90% for almost all immunobiologicals distributed in the public network.^8^ Vaccine-refusal or anti-vaccine groups that spread around the world seem to be few in our environment, but these few can jeopardize the success already achieved by NIP as to infectious diseases control and the improvement of living conditions for the population. Recently, two situations have put the spotlight on the harmful consequences of social networks, which exhaustively disclosed fanciful information about the adverse events of HPV (human papillomavirus) vaccine in Brazilian adolescents and the alleged association of rubella vaccine with microcephaly in infants of Brazilian women possibly infected by the Zika virus. The prompt denial of such rumors and clarification for the population, however, did not prevent the credibility of vaccines from being damaged, which may persist for a long time.^9^ ^,^ ^10^

The anti-vaccine movement and the indecision about/delay in vaccines induce attitudes that put not only an unvaccinated individual’s health at risk, but also everyone’s around them. Epidemics of measles, whooping cough and chickenpox have already been associated with such attitudes, causing unnecessary suffering and increasing public expenditures. Misinformation, inaccurate/insufficient information, myths, pseudoscientific information, time relation to adverse events, lack of memory about previous epidemics severity, lack of credibility in companies that produce vaccines and/or health agencies, religious and philosophical ideologies can be pointed as causes of such attitudes.^11^ ^,^ ^12^ The World Health Organization (WHO) defines “vaccine hesitancy” as a delay in accepting or refusing vaccination despite the availability of vaccine services, and has set up a special board to discuss and establish strategies to cope with this matter: SAGE: Working Group on Vaccine Hesitancy.^13^ Groups that delay acceptance of or refusal to vaccines have complex behavior, are time-varying, pose different regional repercussions, and require continuous monitoring. In addition, levels of discredit are not homogeneous, varying from those who are hesitant (refusing -to or delaying the taking of some vaccines); those who are in-doubt about vaccines, but accept them; and those who totally refuse to take vaccines.^6^ ^,^ ^13^

Children and adults who are not vaccinated as a result of anti-vaccine movements or indecision as to vaccines, and the reasons for such happenings have not been adequately assessed and identified in Brazil yet. Considering physicians (particularly pediatricians) as key factors to maintain the credibility of vaccines and WHO’s recommendations to assess their behavior, the objective of this study was to identify the perception of medical students and physicians on the importance of vaccines and the risks posed by vaccine refusal in a private medical school of the state of São Paulo.

## METHOD

This project was approved by the Ethics Committee of the institution, under protocol no. 1,432,866. All subjects signed a free and informed consent form and the rules of Resolution 466 of the National Health Council (CNS) for research involving human beings, as of December 12, 2012, were complied with.

The best instruments to evaluate trust in vaccine and vaccine refusal are still not well established. To measure hesitation of parents towards vaccines, a tool named The Parent Attitudes about Childhood Vaccines (PACV) was created in 2011,^14^ later on revised and validated by the same group of investigators.^15^ ^,^ ^16^ However, there is still not a valid instrument to conduct this assessment in other populations. For this reason, we chose to create a questionnaire with open and closed questions and statements aimed at evaluating interviewees’ perceptions about the importance of vaccines, the Ministry of Health’s official vaccination schedule, vaccination of the research subject, credibility in NIP, concept of herd protection, vaccine safety, the importance of post-vaccine adverse events, as well as social responsibility and ethical/legal aspects related to vaccination and vaccine refusal.

The cross-sectional study was based on a questionnaire applied in 2016. The convenience sample, formed by 92 subjects selected from a private medical school (São Leopoldo Mandic School of Medicine and Dentistry, Campinas, São Paulo), was divided into two groups: group 1, with 53 medical students (from the first to the fourth grades); and group 2, with 39 doctors (of different specialties, acting as professors or not).

Data collected from the questionnaire were tabulated in the Microsoft Excel program and analyzed statistically in GraphPad Prism software (version 6.0, La Jolla, CA, USA). Response rates in each group were calculated as percentage, using the 95% confidence interval (95%CI), and groups (professors and students) were compared by Fisher’s exact test. The Kruskal-Wallis test was used to compare the accuracy indexes between series. The level of significance was set at 5%.

## RESULTS

Among the 39 physicians in the study, mean age was 46.2±11.0 years, and among the 53 medical students, 22.8±3.3 years. Twenty-four physicians (61.6%) and 23 medical students (43.4%) were males; this difference was not significant (p=0.09). Among students, 10 were in the first year of graduation; 18, in the second year; 18, in the third year; and 7, in the fourth year. Physicians had 16.0±13.5 years of training on average and more than 90% of them were acting as professors. Ten physicians acted with surgical specialties, seven with activities not related to direct patient care, and 22 with clinical specialties. All students and 94.9% of physicians said they had a personal vaccine registry (p=0.177).

Most participants assured that they remembered the last vaccine they received: 47/53 (88.7%) students and 37/39 (94.9%) physicians (p=0,413). They were: influenza (48% of students and 70.3% of physicians); hepatitis A, B or unspecified (17.0% of students and 8.1% of physicians); tetanus or diphtheria/tetanus (8.5% of students and 10.8% of physicians). Other vaccines mentioned as the last received included viral mumps/triple viral (four students), yellow fever (two students), adult acellular triple (two students), meningococcus (one student), and herpes zoster (one professor). Only two students reported having last received the HPV vaccine. One physician and one student reported hepatitis C vaccine. The number of physicians who reported taking influenza vaccine 2015 was larger than students (33/39; 84.5% *versus* 28/53; 52.7%, p=0.0017).

All physicians and 83% of the students (p=0.0091) agreed with the statement that “vaccines protect against potentially fatal diseases”. Most respondents stated that “post-vaccine adverse events are mostly not serious” (92.5% of students and 97.4% of physicians, p=0.390) and that “the benefits of vaccines are much more important (100% of physicians and 98.1% of students, p=1.00). Considering the 14 questions related to basic concepts of vaccination only, the average rate of correctness was 67.7% among students in the fourth year; 72.9% in the first year; 77.0% in the second year; and 79.4% in the third year. This difference was not statistically significant (Kruskal-Wallis test, p>0.05).

The NIP in Brazil was considered efficient and reliable by 88.7% of students and 92.3% of physicians (p=0.728). The number of infectious diseases that can be avoided with vaccines and that are part of the NIP schedule in Brazil for children is unknown by most students (34/53, 64.2%) and by 38.5% (15/39) of physicians (p=0.0201). When asked to cite two vaccines introduced in the basic NIP schedule for free in the last 10 years, 16/39 doctors (41%) and 9/53 (17%) students correctly cited the two vaccines (p=0.0169); 13/39 (33.3%) physicians and 35/53 (45.3%) students (p=0.0029) pointed at least one vaccine correctly.

Of the total, 54.7% of students and 59.0% of physicians stated that they know someone who refused to receive vaccines; and 43.4% of the students and 41.0% of the physicians said they know someone who refuses to vaccinate their children. Physicians who said that they had seen one or more patients who refused to receive vaccines during the six months prior to the application of questionnaires were 19/39 (48.7%). At that, the conduct described by physicians was always to orient them as to the risks and benefits of vaccination/vaccine refusal. The following reasons were considered possible causes for vaccine refusal by physicians and students, respectively: fear of adverse events (89.7 and 94.3%); philosophical reasons (66.7 and 67.9%); religious reasons (51.3 and 67.9%); and lack of knowledge about the severity and frequency of immunopreventable diseases (43.6 and 43.4%). There was no statistical difference between the answers of physicians and students.

Both students (51/53, 96.2%) and physicians (36/39, 92.3%) (p=0.647) believe that, according to the Statute of the Child and Adolescent (ECA), in force in Brazil, a judge can take legal action to ensure a child’s right to vaccination. In the possibility of a child falling ill due to vaccine refusal, and based on the same statute, students (29/53, 54.7%) and physicians (25/39, 64.1%) believe that parents can respond for crime of abandonment, intentional or non-intentional omission, and that this can lead to loss of family power (p=0.399).

Taking into account a patient who refuses to receive a vaccine (or refuses to vaccinate their children), 43.4% of students and 56.4% of physicians considered it reasonable to respect one’s will, but that they would explain the consequences of that choice to the patient. Only a minority would think about compulsory vaccination (9.4% of students and 10.3% of physicians) or would stop assisting these patients (13.2% of students and 15.4% of physicians). Most interviewees stated that it is unethical to disregard the risk to other subjects upon vaccine refusal (83% of students and 61.5% of physicians, p=0.003).

Other responses from students and physicians to questions about vaccines, NIP and ethical matters related to them can be seen in Tables 1 and 2.

**Table 1:** Response from physicians and students to statements related to vaccines and the National Immunization Program.

Statements	Group	Yes n (%)	No n (%)	p-value*
Adverse events are common	Students	9 (17.0)	44 (83.0)	0.5961
	Physicians	9 (23.1)	30 (76.9)	
Before being marketed, vaccines are tested for safety, but not always for effectiveness	Students	8 (15.1)	45 (84.9)	1.0000
	Physicians	5 (12.8)	34 (87.2)	
The Brazilian vaccination schedule protects children early, before exposure to infectious diseases	Students	52 (98.1)	1 (1.9)	1.0000
	Physicians	39 (100)	0 (0)	
Vaccinated children and adults can protect others from infectious diseases	Students	35 (66.0)	18 (34.0)	0.0279
	Physicians	34 (87.2)	5 (12.8)	
Children and adults can receive several vaccines on the same day without harm to vaccine protection	Students	25 (47.2)	28 (52.8)	<0.0001
	Physicians	39 (100)	0 (0)	
Not to vaccinate minors can not only harm them, but also the people around them, since group (herd) immunization is compromised	Students	46 (86.8)	7 (13.2)	0.7541
	Physicians	35 (89.7)	4 (10.3)	
In lower socioeconomic classes, vaccination coverage is lower than in the upper classes	Students	23 (43.4)	30 (56.6)	1.0000
	Physicians	17 (43.6)	22 (56.4)	
The number of vaccines given to children in their first year of life is exaggerated	Students	3 (5.7)	50 (94.3)	0.6346
	Physicians	1 (2.6)	38 (97.4)	

*Fisher’s exact test.

**Table 2:** Answers from physicians and students to ethical questions related to vaccine refusal.

Statements	Group	Yes n (%)	No n (%)	p-value*
Is it ethical to respect only the patient’s will and disregard the health of other individuals?	Students	9 (17.0)	44 (83.0)	0.0300
	Physicians	15 (38.5)	24 (61.5)	
Can a school refuse to receive a child who is not vaccinated because of parents' wishes?	Students	18 (34.0)	35 (66.0)	0.2826
	Physicians	18 (46.1)	21 (53.9)	
Is it defensible (ethically, legally or socially) that parents have total control of the lives of their children, without limitations, deciding on their vaccination?	Students	11 (20.8)	42 (79.2)	0.2306
	Physicians	13 (33.3)	26 (66.7)	
Can a physician refuse to assist families that are against vaccination?	Students	12 (22.6)	41 (77.4)	0.2412
	Physicians	14 (35.9)	25 (64.1)	
Should the physician systematically report families who refuse to vaccinate their children to the Guardianship Council?	Students	18 (34.0)	35 (66.0)	0.0059
	Physicians	25 (64.1)	14 (35.9)	

*Fisher’s exact test.

## DISCUSSION

This exploratory study aimed to identify the perception of medical students and physicians on the importance of vaccines and the risks posed by vaccine refusal in a private medical school. We understand that the number of participants involved is small. The medical school chosen at the time was only in its fourth year of operation; however, obtaining information from 53 out of 435 students enrolled (12.2%) and 39 out of 74 professors (52.7%) allowed us to raise topics to be discussed on the subject.

Having a vaccination record and remembering the latest vaccines received can be considered good indicators of personal protection valuing and trust in vaccines. All students and 94.9% of physicians said that they had a vaccination registry, and most (88.7% of students and 94.9% of physicians) were able to recall the last vaccine received. However, despite belonging to a group at risk for influenza, 15.4% of physicians had not received the vaccine in 2015 and the most commonly reported reason was “lack of interest”. Among students, 47.2% had not received influenza vaccine in 2015 and, although “lack of interest” was included as a relevant justification, the fear of adverse events was cited as the cause of refusal in 16% of occurrences in this group.

The lack of interest of physicians and medical students in a prophylactic measure as important as the influenza vaccine is concerning. In a recent review of 39 European articles, 21 mentions of “fear of adverse events” were found to be the cause of vaccine refusal in the general population and in four among health professionals.^17^

A study carried out with medical students in São Paulo evaluated the adherence to influenza vaccination in the pandemic (2010) and post-pandemic period (2011) and showed a drop from 92 to 41% of vaccine coverage from one year to the other, suggesting that, outside pandemic periods, adherence in this population is low.^18^ A survey conducted with resident doctors of the specialty Family Medicine in Seoul, South Korea, pointed out that although coverage for influenza vaccine was good (83.0%), the knowledge about all vaccines recommended for health professionals by the local infectious society was very low (9.9%).^19^

A health professional, as a result of their job, is exposed to infectious agents that can lead them to develop (themselves or people they get in contact with - patients and relatives) infectious diseases. Vaccination of health professionals, however, has been described as suboptimal in several locations, which leads to a discussion on the need for compulsory vaccination for this group.^20^

Even the NIP being considered reliable, 17% of students do not acknowledge that vaccines protect against potentially life-threatening diseases, and 64.5% of students and 38.5% of physicians are unaware of all the vaccines part of the official vaccination schedule. Not knowing the potential severity of vaccine-preventable diseases or not knowing the vaccines available at no cost to the population may determine nonconcern with the potential risk of such diseases, and with the individual and collective importance of vaccines among health professionals. The success of vaccine coverage has the correct and adequate knowledge by the health professional (and medical student) about the importance of vaccines as determining factor.^21^

Although most physicians and students recognize that vaccinated individuals can protect others from infectious disease, we found 18/53 (34%) students who do not acknowledge the importance of herd protection in controlling infectious diseases. The recent outbreak of measles in the United States, in Brazil and still persisting in Europe could have been minimized had herd protection been sufficient in the affected populations. Since vaccination coverage cannot reach 100% of the population, immunizing most subjects makes it more difficult to disseminate the virus to people with actual contraindications to the vaccine or those who, even though were vaccinated, did not obtain adequate protection.^22^

Among other factors, the simultaneous application of several vaccines has been considered one of the reasons for individuals to have queries about or refuse to receive vaccines.^11^ ^,^ ^17^ ^,^ ^23^ Even though recognized as effective by all physicians, our study pointed several vaccines being applied on the same day caused concern among students as to efficacy of this practice: more than half of them considered the possibility that this could incur in prejudice to vaccine protection, ignoring the safety and efficacy of this practice, which demands a thorough discussion of this topic.

Ethical matters related to vaccine refusal^12^ ^,^ ^24^ ^,^ ^25^ and the possibility of using legal measures, based on the ECA, to convince parents to vaccinate their children have motivated discussions between physicians and lawmakers and are not fully understood by physicians and students yet. Although most of the study population considered unethical to refuse vaccines, while disregarding the health of others, besides the fact that parents have power of decision over the life of their children without limitations and decide on vaccine application without taking the collective into account, students are more likely than physicians to systematically report families who refuse to vaccinate their children to the Guardianship Council (p=0.059).

Vaccination of children involves balancing the autonomy of parents in deciding whether to immunize their children or not and the benefits of mass vaccination campaigns to public health.^3^ The ECA states that it is the family’s duty to ensure the rights to health of children and adolescents, which includes routine vaccination.^26^ In addition, family members who oppose to the vaccination of their children can seriously impair the doctor-patient relationship, in a way that allows the physician to stop providing care for the patient (Medical Code of Ethics, art. 36).^27^

The great penetration and easy access to social media has promoted an enormous amount of information - not always correct - on the safety (or unsafety) of vaccines, their effectiveness, efficacy, risks, etc., based on philosophical, political and religious grounds. The proliferation of information of this kind may jeopardize the success of vaccination programs. Knowledge by the health professional with the competence and confidence to clarify the theme can minimize this risk.

In 2016, the concern with vaccine-refusal groups and their influence on the general population and vaccine coverage rates led the WHO, the American Academy of Pediatrics, and the American Academy of Medicine to produce manuals and guides to help physicians cope with this new reality.^28^ ^,^ ^29^

An important limitation of this study was the sample size; in addition to being small, it was chosen for convenience and may not accurately represent the groups studied. For this reason, it is not possible to generalize the findings, but, for the group studied, conclusion is that medical students and physicians have queries about vaccination schedule, vaccine safety, herd protection and ethical aspects of vaccine refusal. Such queries can lead to unpreparedness when addressing the issue of vaccine denial, which has been rising worldwide.

Considering that adequate immunization of health professionals is recognized as the best form of protection against infectious risk,^30^ the vaccination of this group and their beliefs should also be evaluated in the context of vaccine refusal. Knowing the possible flaws in concepts among this population will be useful when introducing new contents in the teaching programming of immunizations and prevention of infectious diseases. Emphasizing discussion on vaccination and its importance in medical school curriculi can empower the future physician for the decision-making process regarding vaccination, approaching vaccination itself, and vaccine refusal in an ethical way, thus maintaining the success obtained with vaccination programs.
